# Subanesthetic ketamine infusions for the treatment of children and adolescents with chronic pain: a longitudinal study

**DOI:** 10.1186/s12887-015-0515-4

**Published:** 2015-12-01

**Authors:** Kathy A. Sheehy, Elena A. Muller, Caroline Lippold, Mehdi Nouraie, Julia C. Finkel, Zenaide M N Quezado

**Affiliations:** Divisions of Anesthesiology and Pain Medicine, The Sheikh Zayed Institute for Pediatric Surgical Innovation, Children’s Research Institute, Children’s National Health System, George Washington University School of Medicine and Health Sciences, Washington, USA; Center for Sickle Cell Disease, Department of Internal Medicine, Howard University, Washington, USA; Center for Neuroscience Research, Children’s Research Institute, Children’s National Health System, Washington, USA

## Abstract

**Background:**

Chronic pain is common in children and adolescents and is often associated with severe functional disability and mood disorders. The pharmacological treatment of chronic pain in children and adolescents can be challenging, ineffective, and is mostly based on expert opinions and consensus. Ketamine, an N-methyl-D-aspartate receptor antagonist, has been used as an adjuvant for treatment of adult chronic pain and has been shown, in some instances, to improve pain and decrease opioid-requirement. We examined the effects of subanesthetic ketamine infusions on pain intensity and opioid use in children and adolescents with chronic pain syndromes treated in an outpatient setting.

**Methods:**

Longitudinal cohort study of consecutive pediatric patients treated with subanesthetic ketamine infusions in a tertiary outpatient center. Outcome measurements included self-reported pain scores (numeric rating scale) and morphine-equivalent intake.

**Results:**

Over a 15-month period, 63 children and adolescents (median age 15, interquartile range 12–17 years) with chronic pain received 277 ketamine infusions. Intravenous administration of subanesthetic doses of ketamine to children and adolescents on an outpatient basis was safe and not associated with psychotropic effects or hemodynamic perturbations. Overall, ketamine significantly reduced pain intensity (*p* <0.001) and yielded greater pain reduction in patients with complex regional pain syndrome (CRPS) than in patients with other chronic pain syndromes (*p* = 0.029). Ketamine-associated reductions in pain scores were the largest in postural orthostatic tachycardia syndrome (POTS) and trauma patients and the smallest in patients with chronic headache (*p* = 0.007). In 37 % of infusions, patients had a greater than 20 % reduction in pain score. Conversely, ketamine infusions did not change overall morphine-equivalent intake (*p* = 0.3).

**Conclusions:**

These data suggest that subanesthetic ketamine infusion is feasible in an outpatient setting and may benefit children and adolescents with chronic pain. Further, patients with CRPS, POTS, and a history of trauma-related chronic pain are more likely to benefit from this therapeutic modality.

## Background

Chronic pain, defined as pain lasting for more than 3 months, is common in children and adolescents and its prevalence varies according to pain location and associated primary disease [1–4]. Clinicians treating children and adolescents with chronic pain recognize that it can pose diagnostic and therapeutic challenges and children with chronic pain can have significant physical disabilities, decreased mobility, sleep disturbances, and mood disorders [5–8]. Furthermore, pain-related interference with school attendance and daily activities and pain-related emotional disturbances can be associated with increased risks of developmental stagnation and suicidal ideation [6, 7, 9, 10]. Given the biological, psychological, and social consequences of chronic pain in children and adolescents, its clinical evaluation requires exploration of these various domains and its therapy a multidisciplinary approach [11, 12].

In many children and adolescents, the pharmacologic treatment of chronic pain requires a combination of opioids, anticonvulsants, and antidepressants [13]. However, despite administration of drugs with various mechanisms of action, the treatment of chronic pain in children and adolescents can remain ineffective [13, 14]. Further, the choice of pharmacological agents to treat children and adolescents with chronic pain is predominately based on expert opinions, studies showing efficacy of given therapies in adults, and on practitioner’s consensus [15–17]. Further, as randomized clinical trials of effective therapies are lacking, the approach to treat pain in children and adolescents often involves trial and error. Therefore, it is imperative that effective therapies are developed to improve the health outcomes of children and adolescents with chronic pain.

Researchers have shown that activation of the N-methyl-D-aspartate (NMDA) receptor increases excitatory transmission in afferent pathways, contributes to sensitization of nociceptive neurons, and is involved in induction and maintenance of central sensitization, thus playing a role in the development of chronic pain [18, 19]. For this reason, ketamine, an NMDA receptor antagonist, has been investigated as an adjuvant for the treatment of chronic pain syndromes. There is some evidence, albeit not from large randomized clinical trials, suggesting that ketamine decreases pain intensity and reduces opioid requirements when used as an adjuvant to therapy of chronic and acute pain in adults [20–32]. In small clinical trials and case-series of adult patients with complex regional pain syndrome (CRPS), ketamine yields analgesia, which in some cases is reported to last for up to 11 weeks after treatment [29, 30, 32, 33]. While the use of ketamine for chronic pain has been studied much less frequently in children than in adults, its use has shown some promise in controlling pain in pediatric patients with cancer [34]. In a phase I trial, five out of 12 adolescents with chronic pain had significant improvement in pain scores and two had resolution of pain after a 2-week oral ketamine treatment [23]. Therefore, while large randomized clinical trials are lacking, there is some evidence to suggest that ketamine might have a role in the treatment of chronic pain in children and adolescents.

This study was conducted to examine the administration of subanesthetic doses of ketamine to treat children and adolescents with chronic pain on an outpatient setting and to determine its effect on reported pain intensity and opioid intake.

## Methods

This study was performed in compliance with the Helsinki Declaration. The study protocol was approved by the Children’s National Health System Institutional Review Board. A waiver of informed consent for this study was also approved by the Children’s National Health System Institutional Review Board, as the data examined had been collected during the clinical care of the patients and was de-identified after its collection. We conducted an observational longitudinal cohort study and examined the medical records of all patients diagnosed with chronic pain syndromes who had received subanesthetic doses of ketamine in an outpatient setting. At the outpatient tertiary pain center, diagnoses of chronic pain syndromes were made following criteria from the International Association for the Study of Pain [35, 36].

We included all patients diagnosed with chronic pain who received subanesthetic ketamine infusions in the outpatient clinic from January 2013 to April 2014. Patients with the diagnosis of acute pain were excluded. For the purpose of this investigation, patients with CRPS types 1 and 2 were included jointly in the CRPS group. Patients with chronic pain syndromes other than CRPS are referred to as having other chronic pain syndromes.

### Ketamine administration

A multidisciplinary pain management team directed pain therapy and administration of subanesthetic doses of ketamine (here defined as doses lower than 1 mg/kg) as clinically indicated [37]. Informed consent for ketamine administration was obtained from parents, guardians, or patients (18-year-old or older) at the time of its administration as part of their clinical care. Each ketamine treatment consisted of infusions at doses of 0.1–0.3 mg/kg/h that lasted for 4–8 h per day, up to a maximum of 16 h in total over a maximum of three consecutive days. Ketamine was administered by a registered nurse and an anesthesiologist was available throughout the infusions. Heart rate, blood pressure, and pulse oximetry were monitored throughout ketamine infusions. Pain scores, based on a numeric rating scale (0 = no pain and 10 = worst pain) [38], were recorded before and after each infusion while oral morphine-equivalent opioid intake was recorded before each infusion and up to 1 month after ketamine administration during follow-up visits. For patients undergoing repeated ketamine treatments, a minimum inter-treatment interval of 4 weeks was observed.

### Outcomes

The primary outcome was change in pain scores measured with a numeric rating scale. Pain scores were measured both before and after each ketamine infusion. The secondary outcome was oral morphine-equivalent opioid intake. Data collected included demographics (sex, age, and race), medical history, heart rate, blood pressure, pulse oximetry, and reports of psychotropic effects, nausea, vomiting, and changes in sleep pattern. Oral morphine-equivalent opioid (per body weight) was calculated as previously described [39] using the conversion tool available at the website www.globalrph.com/narcoticonv.htm.

We also determined the percentage of ketamine infusions that resulted in a clinically meaningful change in pain scores and opioid intake. We defined clinically significant change as a greater than 20 % reduction in pain scores and a greater than 20 % reduction in morphine-equivalent intake. A 20 % reduction in pain scores is greater than the 12.5 % decrease advocated by others as a minimally clinically significant difference in pain scores for adolescents with chronic pain [40].

### Statistical analysis

We analyzed the data by first considering each ketamine infusion and then by considering each ketamine treatment (one to three infusions over three consecutive days). For each infusion, the effect of ketamine on pain scores (after vs. before) and morphine-equivalent intake was assessed by paired t-test. In a separate repeated measure analysis, we assessed the effect of ketamine on pain scores according to different age groups, gender, race, number of treatments, number of infusions, pain diagnosis (CRPS vs. other chronic pain syndromes), comorbidities, primary disease and pain location using subgroup analysis and assessing the interaction between treatment effect and subgroups. Next, we assessed the effect of significant predictors of pain reduction in a multivariate model in which the p value (beta) for interaction represents the heterogeneity of ketamine effect by subgroup (and its direction). That is, a significant p value for interaction between ketamine effect and CRPS indicated that effect of ketamine in pain reduction is different between CRPS and other chronic pain syndromes groups, and a negative beta value showed that ketamine caused more pain reduction in the CRPS group. Variables with *p* ≤0.2 were entered into the multivariate model and the final model was developed using a stepwise backward approach. For the analysis of percent changes in pain scores and opioid intake, we calculated these changes by subtracting post-infusion from pre-infusion values and dividing the difference by pre-infusion values of respective outcomes.

For each ketamine treatment (one to three daily infusions) we compared the pain score and morphine-equivalent intake reported before the first with that obtained after the last infusion using paired t-test. As discussed earlier, the relationships between the effect of ketamine and age, gender, race, number of ketamine treatments, CRPS, pain location, comorbidities, and associated clinical diagnoses were assessed.

## Results

### Patients and clinical findings

Between January 2013 and April 2014, 63 patients with chronic pain syndromes received a total of 111 treatments delivered over 277 ketamine infusions (Table 1). Indications for ketamine administration included 1) requirement of escalating doses of opioid associated with non-tolerated side effects (excessive sedation or constipation) or 2) lack of improvement in pain intensity and/or disabilities with other standard treatment modalities (anticonvulsants and/or antidepressants).

**Table 1:** Number of treatments and infusions of sub-anesthetic ketamine administered to 63 children and adolescents with chronic pain syndromes in an outpatient tertiary pediatric care referral center^a^

Treatment	Number of patients receiving ketamine infusions			Total
	First	Second	Third	
First	63	61	37	161
Second	26	23	13	62
Third	12	12	6	30
Fourth	7	7	4	18
Fifth	3	3	0	6
Total	111	106	60	277

^a^For each treatment, two to three daily infusions were planned to amount to a maximum of 16-h of infusion time

Patient demographics and pain diagnoses and location are shown in Table 2. The majority of patients diagnosed with chronic pain syndromes who were treated with ketamine in this study were Caucasian females. Thirty-seven percent (23 out of 63) of patients had CRPS (Type 1, *N* = 21 and Type 2, *N* = 2), and 63 % (40 out of 63) had other chronic pain syndromes including chronic headache (13 %) and fibromyalgia (5 %). The predominant primary pain location was the lower extremities (35 %) followed by generalized pain (16 %), back pain (16 %), chronic headaches (13 %), upper extremity (8 %), and abdominal pain (6 %). Most patients (68 %) reported no secondary pain location. Associated clinical diagnosis included psychiatric/psychological disorders in 23 % (anxiety, depression, bipolar disorder, and autism spectrum disorder), history of trauma in 10 %, postural orthostatic tachycardia syndrome (POTS) in 10 %, diabetes mellitus in 7 %, malignancy in 7 %, and sickle cell disease in 5 % of the patients (Table 3).

**Table 2:** Demographic characteristics of 63 children and adolescents with chronic pain syndromes treated with ketamine in an outpatient tertiary pediatric care referral center^a^

Characteristic	N (%)
Sex	
Female	45 (71 %)
Male	18 (29 %)
Age, median (IQR)	15 (12–17)
Race/Ethnicity	
Caucasian	46 (63 %)
African American	14 (22 %)
Hispanics	2 (3 %)
Asian/Pacific Islander	1 (2)
Pain diagnosis	
CRPS	23 (37 %)
Other Chronic Pain syndromes	40 (63 %)
Chronic headache	8 (13 %)
Fibromyalgia	3 (5 %)
Pain location	
Lower extremity	22 (35 %)
Generalized	10 (16 %)
Back	10 (16 %)
Head	8 (13 %)
Upper extremity	5 (8 %)
Abdomen	4 (6 %)
Chest	3 (5 %)
Testicles	1 (2 %)

^a^Characteristics are those at the time of first ketamine infusion. *N* indicates number, IQR interquartile range, and CRPS indicates complex regional pain syndrome and include CRPS type 1 (*N* = 21) and type 2 (*N* = 2)

**Table 3:** Associated diagnosis in 63 children and adolescents with chronic pain syndromes treated with ketamine in an outpatient tertiary care pediatric center^a^

Associated diagnoses	N (%)
Psychiatric/psychologic disorders	14 (23 %)
Trauma	12 (10 %)
POTS	6 (10 %)
Diabetes mellitus	4 (7 %)
Malignancy	4 (7 %)
Sickle cell	3 (5 %)
Neurofibromatosis 1	2 (3 %)
Lyme disease	1 (2 %)
Chemotherapy-induced neuropathy	1 (2 %)

^a^ Psychiatric/psychiatric disorders recorded included anxiety, depression, bipolar disorder, and autism spectrum disorder. POTS indicates postural orthostatic tachycardia syndrome

### Primary outcome

**Fig. 1:**
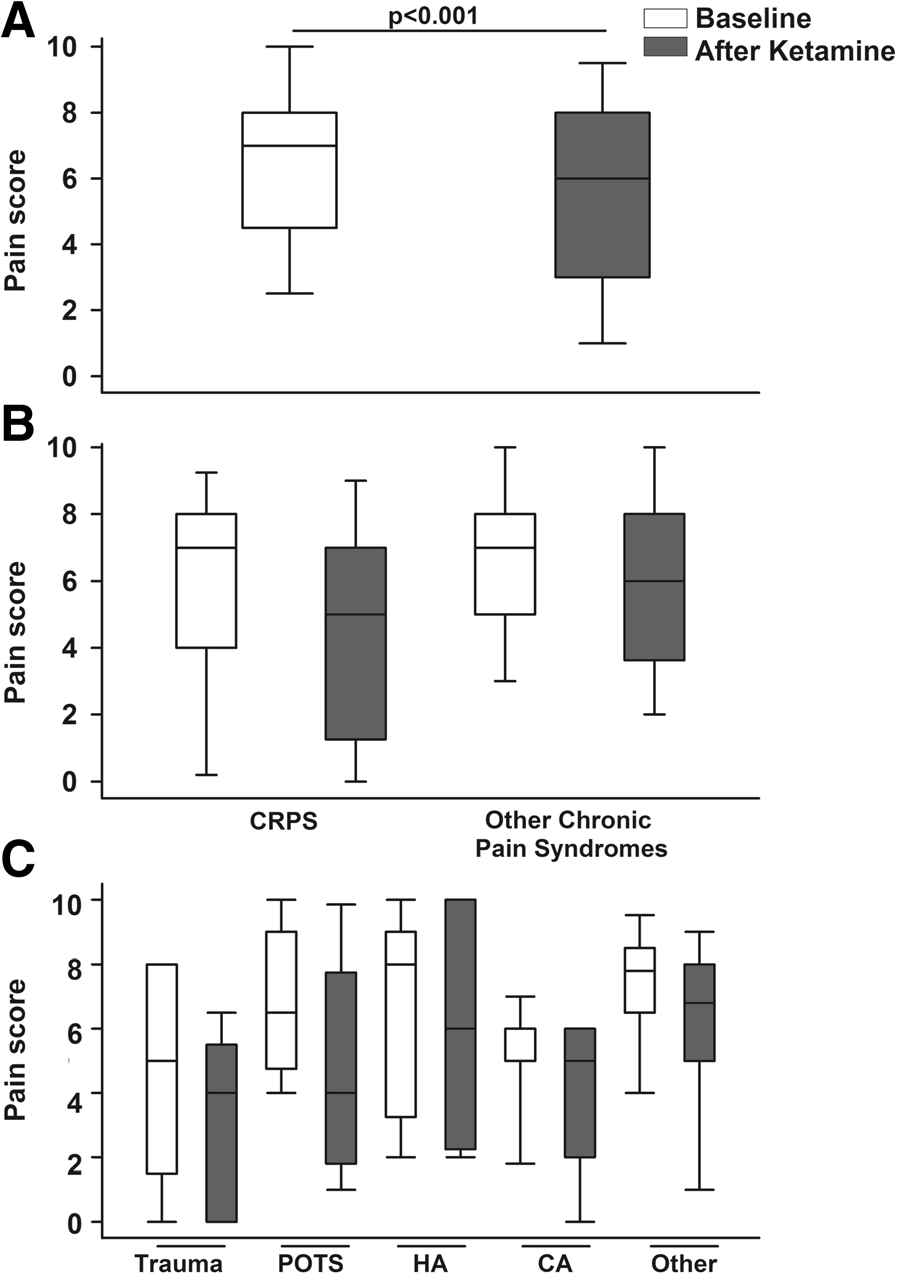
Effect of ketamine infusions on pain scores (numeric rating scale) in children and adolescents with chronic pain. The *box plots* show the data’s median and interquartile range and the whiskers the 5^th^ and 95^th^ percentiles. **a** Box plots of pain scores at baseline (*white*) and after ketamine infusions (*gray*) in all patients. Subanesthetic ketamine infusions in an outpatient setting significantly reduced pain scores (*p* <0.001). **b** Effect of ketamine infusions on pain scores according to pain diagnoses of complex regional pain syndrome (CRPS) or other chronic pain syndromes. The reductions in pain scores after ketamine infusions were significantly greater in patients with CRPS than in patients with other chronic pain syndromes (*p* = 0.029). **c** *Box plots* of pain scores at baseline (*white*) and after ketamine infusion (*gray*) for patients with history of trauma, postural orthostatic tachycardia syndrome (POTS), chronic headache (HA), malignancy (CA), and other associated condition (sickle cell, diabetes). The reductions in pain scores after ketamine infusions were the greatest in trauma and POTS patients and the lowest in patients with chronic headache (*p* = 0.007 for overall difference)

We first analysed the effect of ketamine on pain, considering each infusion (each day) and compared pain scores after and before each infusion (*N* = 277). Subanesthetic doses of ketamine significantly reduced pain scores overall (*p* <0.001) after each infusion, Fig. 1. Interestingly, the effect of ketamine on pain scores varied according to primary pain diagnosis and associated clinical conditions. Specifically, ketamine infusions yielded greater pain score reductions in patients with CRPS than in patients with other chronic pain syndromes (*p* = 0.029, Fig. 1b). Further, pain score reductions after ketamine infusions were the greatest in trauma and POTS patients and the lowest in patients with chronic headache (*p* = 0.007 for overall difference, Fig. 1C). We then conducted a multivariate analysis to identify predictors of the effect of ketamine in reducing pain scores. Using data from each ketamine infusion, older age, chemotherapy-induced neuropathy, and CRPS, were significant predictors of greater pain score reductions (Table 4). There was no impact of age, sex, race, number of ketamine treatment or infusion on the beneficial effect of ketamine in reducing pain scores (all *p* ≥0.1).

**Table 4:** Predictors of pain score reduction after sub-anesthetic doses of ketamine infusions in children and adolescents with chronic pain syndromes treated in an outpatient tertiary care pediatric center ^a^

Variable	*P* value	Beta
Multivariate analysis after each infusion		
Older age	0.004	−0.1
Chemotherapy neuropathy	0.006	−3.1
Complex regional pain syndrome	0.018	−0.7
Multivariate analysis after each treatment		
Older age	<0.001	−0.2
Chemotherapy neuropathy	<0.001	−7.3
Complex regional pain syndrome	<0.001	−2.1

^a^In the multivariate model, the *P* value for interaction represents the heterogeneity of ketamine effect by subgroup and the beta value indicates its direction. That is, a significant *P* value for interaction between ketamine effect and complex regional pain syndrome (CRPS) indicated that effect of ketamine in pain reduction is different in CRPS than other chronic pain syndromes. A negative beta value indicates that ketamine caused greater pain reductions in patients in the CRPS group compared to other chronic pain syndromes. When analyzing each ketamine treatment (one to three infusions), the pain score after the last infusion was compared with the one before the first infusion

We then examined the effect of ketamine treatments (one to three consecutive daily infusions amounting to a total of 16-hour infusions, Table 1) on pain scores and found that ketamine treatments significantly decreased pain scores (−1.6 ± 0.24, mean change ± standard error of the mean, SEM, *p* <0.001). Similar to the findings after each daily infusion, ketamine treatments also yielded significantly greater decreases in pain scores in patients with CRPS compared to those with other chronic pain syndromes (*p* = 0.005). Also similar to the findings after each ketamine infusion, older age, chemotherapy-induced neuropathy, and CRPS were significant predictors of greater ketamine-associated reductions in pain scores (Table 4).

### Secondary outcomes

With regards to the effect of ketamine on opioid intake, after each infusion, ketamine did not change oral morphine-equivalent intake (−0.03 ± 0.031, mean change ± SEM, *p* = 0.3) compared to baseline doses. Similarly, ketamine treatments (up to three daily infusions) did not reduce oral morphine-equivalent intake (−0.1 ± 0.05 mg/kg/day, *p* = 0.17). In addition, there was no impact of pain diagnosis, pain location, or associated clinical diagnosis on the effect of ketamine on oral morphine-equivalent intake (all *P* ≥0.8).

During ketamine administration (infusions or treatments), no psychotropic side-effects, hallucinations, nausea, vomiting, or changes in sleep pattern were reported by any patient. Further, no hemodynamic changes or arrhythmias were observed during infusions.

### Frequency of significant reduction in pain score and morphine-equivalent intake

In order to determine whether ketamine infusions yielded clinically significant changes in pain scores, we examined the frequency of infusions that yielded 20 % or greater pain score reductions and 20 % or greater reductions in morphine-equivalent intake compared to baseline levels. In 37 % of infusions (*N* = 99 out of 277), ketamine yielded a greater than 20 % reduction in pain scores compared to baseline. The frequency of ketamine infusions that yielded a greater than 20 % reduction in pain scores did not vary by age (*p* = 0.6), sex (*p* = 0.9), race (*p* = 0.3), ketamine treatment (*p* = 0.4) or infusion numbers (*p* = 0.5), pain diagnosis (*p* = 0.17), associated clinical diagnosis (*p* = 0.8), or pain location (*p* = 0.7).

When we examined the changes in opioid intake considering only those infusions administered to patients taking opioids, we found that in 12 % of infusions (*N* = 9 out of 77), patients had greater than 20 % reduction in morphine-equivalent intake. In addition, the frequency of greater than 20 % reduction in morphine-equivalent intake was unaffected by age (*p* = 0.6), race (*p* = 0.3), comorbidities (*p* = 0.12), or associated conditions (*p* = 0.3). In contrast, the frequency of ketamine infusions that yielded greater than 20 % reduction in morphine-equivalent intake was significantly lower in males than in females [0.2 (0.1, 0.7), odds ratio (95 % confidence interval, CI), *p* = 0.013]. While there were no overall effect of ketamine on opioid intake, among patients taking opioids, the frequency of ketamine infusions that yielded a greater than 20 % reduction in morphine-equivalent intake was significantly higher in patients with CRPS than in patients with other chronic pain syndromes [3.3 (1.3, 8.4), odds ratio (95 % CI), *p* = 0.014]. The frequency of ketamine infusions that yielded a greater than 20 % reduction in morphine-equivalent intake also varied by pain location (overall, *p* = 0.021), specifically it was 23 % for patients with abdominal pain, 22 % for pain in the lower extremities, 20 % in the upper extremities, 5 % in the back, 4 % for global pain, and 0 % in the chest and head. Lastly, the frequency of a greater than 20 % reduction in morphine-equivalent intake varied by ketamine treatment (*p* = 0.035), specifically, the frequency was 21 % for the first treatment, 4 % for the second and 0 % for the third to fifth treatments.

## Discussion

The rational for using ketamine, an NMDA receptor antagonist, to treat chronic pain including CRPS includes the evidence that activation of the NMDA receptor is involved in the pathobiology of central sensitization and chronic and neuropathic pain [18, 19, 41]. NMDA activation is believed, at least in part, to underlie increases in excitatory transmission in afferent pathways in the central nervous system and contribute to hypersensitivity and increased pain [18, 19, 41]. However, most studies have been conducted in adults and definitive data supporting the efficacy of ketamine for the treatment of CRPS or other types of chronic pain is lacking [20]. In fact, based on reviews of existing studies, European guidelines rate the evidence to support the treatment of CRPS with ketamine as level 3 or moderate [42, 43].

Clinicians facing the challenge of treating patients, especially children and adolescents, with chronic pain and/or CRPS, who despite treatment with a number of available therapies remain in pain, are left with very few alternatives. Here, we examined the effect of repeated subanesthetic ketamine infusions on pain intensity and opioid intake in children and adolescents with chronic pain. In this longitudinal outpatient cohort study, we found that subanesthetic ketamine administration to children and adolescents with chronic pain in an outpatient setting was safe and not associated with undesirable psychotropic effects or hemodynamic changes. Overall, ketamine yielded significant decreases in pain intensity and greater reductions in pain scores were observed in adolescents with CRPS compared with other types of chronic pain. Interestingly, when we examined associated clinical diagnoses, we showed that ketamine yielded the greatest reductions in pain scores in patients with history of trauma and of POTS and the lowest in patients with chronic headache. This study is informative and can be helpful for the design of future controlled randomized studies to evaluate the optimum ketamine administration regimen to treat children and adolescents with chronic pain.

The finding that ketamine had a greater analgesic effect in patients with CRPS compared to other chronic pain syndromes is in keeping with adult studies showing that ketamine is associated with significant pain relief in CRPS patients, an effect, which can be sustained [29, 30, 32, 33]. Here, using multivariate analysis, we found that the diagnosis of CRPS was a significant predictor of a beneficial ketamine effect in reducing pain scores. One limitation of this study is that we did not evaluate the long-term effects of ketamine infusion on pain scores or on functional capacity that might have been associated with the improvement in pain scores. However, this deficiency will be addressed in future studies not only to determine the duration of pain improvement, but also the effect on functional performance of children and adolescents treated with ketamine. Nevertheless, in this sample of patients, it appears that, akin to adults, children and adolescents with CRPS have greater reductions in pain scores compared to other chronic pain syndromes.

The finding that trauma patients had greater reductions in pain scores after ketamine administration is not surprising as many patients with CRPS have a history of trauma or surgery preceding the development of CRPS. In contrast, the finding that patients with POTS also had greater reduction in pain scores compared to other chronic pain syndromes is intriguing and should be further studied. To our knowledge, this is the first report of the use of ketamine in the POTS patient population. Given that there is emerging evidence that in patients with POTS, chronic pain can be a frequent manifestation, pose significant morbidity, and be associated with poor response to conventional analgesic therapy [44, 45], ketamine could be further explored as an alternative therapeutic approach.

The evaluation of clinically meaningful changes in pain intensity is important for the evaluation of pain therapies. In adults with chronic pain, researchers estimate that on average, a reduction of approximately two points (average decrease −1.74) or of 30 % in pain scores (numeric rating scale from 0 to 10) represents a clinically relevant and meaningful difference [46]. While large studies in children are lacking, researchers showed that in post-operative settings, the minimum clinically significant difference is estimated to be a reduction of approximately 1 point on the numeric rating scale [47]. Others have shown that in children with chronic pain, reductions of 1 point and of 12.5 % on a numeric rating scale (from 0 to 10) meet criteria for minimally significant differences [40]. Here, we found an average reduction of −1.6 points in pain scores and noted that in 37 % of ketamine infusions, patients had a greater than 20 % decrease in pain scores. Therefore, our findings suggest that ketamine treatment of children and adolescents with chronic pain is associated with clinically significant reductions in pain intensity.

We note that in almost 25 % of our patients, there were associated psychiatric/psychological disorders and it is unclear whether the development of chronic pain preceded these disorders. Ketamine has been shown to have a significant and sustained antidepressant effect as described in adults with major depression [48, 49]. This antidepressant effect is observed after only one dose of 0.5 mg/kg of ketamine administered over 40 min and in some patients it lasts for weeks [48, 49]. While we did not investigate whether ketamine had any effect on mood in this sample of adolescents with chronic pain, it is conceivable that the beneficial effect of ketamine on pain scores could in part be associated with possible improvement in mood.

Interestingly, we observed that ketamine improved pain scores but did not have a significant effect on opioid consumption. We consider two possibilities to explain this apparent inconsistency 1) the study lacked power to determine the effect of ketamine in opioid use given that in only 77 of the 277 infusions, patients were taking opioid and 2) the use of opioid was measured by the prescribed doses during follow up appointments at varying intervals. We also note that while adult studies report a significant incidence of psychotropic effects in the course of ketamine infusions [26], at the doses used in our cohort, ketamine was not associated with psychotropic effects. One can postulate that these discrepant results could be related to differences in doses used on a per kg/h basis, duration of infusions, or differences in pharmacokinetics comparing children and adults. For example, pharmacokinetic and pharmacodynamic studies indicate that children have a shorter context-sensitive half-time and lower sensitivity to ketamine compared to adults [50, 51]. These apparent discrepancies will be further explored in future studies of the use of ketamine in children and adolescents with chronic pain.

## Conclusion

This report suggests that in an outpatient setting, the treatment of chronic pain in children and adolescents with repeated infusions of ketamine is feasible and safe. The findings also suggest that ketamine might have a differential beneficial effect in patients with certain chronic pain syndromes. However, the results must be interpreted with caution given the limitations of the study, including lack of a control treatment group, and the heterogeneous nature of the patient population studied. Nonetheless, despite its limitations, this study raises a number of hypotheses that warrant further testing and it can inform the design of future studies to determine the long-term effects and the optimum ketamine regimen in children and adolescents with CRPS. In addition, given the increased recognition that POTS can be associated with chronic pain that in some patients is refractory to therapy, ketamine should be investigated as a possible adjuvant in therapeutic armamentarium to treat pain in those patients.

## Abbreviations

CI: confidence interval
CRPS: complex regional pain syndrome
NMDA: N-methyl-D-aspartate
POTS: postural orthostatic tachycardia syndrome
SEM: standard error of the mean
